# Reference Frame Unification of IMU-Based Joint Angle Estimation: The Experimental Investigation and a Novel Method

**DOI:** 10.3390/s21051813

**Published:** 2021-03-05

**Authors:** Chunzhi Yi, Feng Jiang, Chifu Yang, Zhiyuan Chen, Zhen Ding, Jie Liu

**Affiliations:** 1 School of Mechatronics Engineering, Harbin Institute of Technology, Harbin 150001, China; chunzhiyi@hit.edu.cn (C.Y.); cfyang@hit.edu.cn (C.Y.); 16b908060@stu.hit.edu.cn (Z.D.); 2 School of Computer Science and Technology, Harbin Institute of Technology, Harbin 150001, China; 3 School of Computer Science, University of Nottingham Malaysia Campus, Semenyih 43500, Malaysia; zhiyuan.chen@nottingham.edu.my; 4 AI Research Institute, Harbin Institute of Technology, Shenzhen 518055, China; jieliu@hit.edu.cn

**Keywords:** inertial measurement unit, joint angle estimation, reference frames, dirft correction

## Abstract

Inertial measurement unit (IMU)-based joint angle estimation is an increasingly mature technique that has a broad range of applications in clinics, biomechanics and robotics. However, the deviations of different IMUs’ reference frames, referring to IMUs’ individual orientations estimating errors, is still a challenge for improving the angle estimation accuracy due to conceptual confusion, relatively simple metrics and the lack of systematical investigation. In this paper, we clarify the determination of reference frame unification, experimentally study the time-varying characteristics of reference frames’ deviations and accordingly propose a novel method with a comprehensive metric to unify reference frames. To be specific, we firstly define the reference frame unification (RFU) and distinguish it with drift correction that has always been confused with the term RFU. Secondly, we design a mechanical gimbal-based experiment to study the deviations, where sensor-to-body alignment and rotation-caused differences of orientations are excluded. Thirdly, based on the findings of the experiment, we propose a novel method to utilize the consistency of the joint axis under the hinge-joint constraint, gravity acceleration and local magnetic field to comprehensively unify reference frames, which meets the nonlinear time-varying characteristics of the deviations. The results on ten human subjects reveal the feasibility of our proposed method and the improvement from previous methods. This work contributes to a relatively new perspective of considering and improving the accuracy of IMU-based joint angle estimation.

## 1. Introduction

Estimating angles of human limb joints plays a fundamental role in obtaining human kinestate and assessing human ability, which is involved in various fields ranging from rehabilitation to robotics, from industry to household. Clinicians observe the joint rotation conditions of lower-limb joints to diagnose the ongoing progression of gait-related diseases, such as muscle dystrophy, joint injuries and stroke, etc. [1,2,3,4,5], based on which further treatment like surgical intervention, recovery or rehabilitative therapy [6,7] will be applied. As for robotics, joint angle estimation contributes to the robots’ awareness of human kinestate and thus results in a smart and accurate assistive and/or rehabilitative action during physical human-robot interaction scenarios [8,9]. Moreover, the angle estimation-involved techniques enable the assessment of human ability. For example, studies predicted risk-related information based on the precepted joint angle information, like [10] for fall prevention, ref. [11] for working safety assessment. Other studies utilized joint angles to assess motor-related information, like [12] for metabolic cost estimation, ref. [13] for mobility assessment for sports. Given that joint angle estimation functions importantly in multiple fields, some problems remain to be solved for the mobile, wearable sensor-enabled joint angle estimation for a take-home or easy-to-integrate usage.

Traditionally, optical motion capture (OMC) is accepted as the “gold standard” for obtaining human joint angles. When leveraging OMC, reflective markers are firstly placed on analytical landmarks on human segments following the principles proposed in [14,15]. The trajectories of reflective markers that represent human motions can be captured by infrared cameras. After lower-pass filtering, the coordinate frames of segments are reconstructed through “pose estimation” process through a global optimization method [16,17]. The relative orientation among the body-fixed coordinate frames of adjacent segments is then fed to the inverse kinematics model in order to calculate joint angles of interest [18]. Although with the capability of providing accurate measurements of joint angles, OMC suffers from its obvious limitations, such as costly facilities, requirement of controlled laboratory settings, trained staff, the inability of real-time application and limited capture volume. These flaws may impede OMC’s broad usage out of laboratories, especially under the current trend of take-home healthcare. Inertial measurement units (IMUs) provide an alternative way due to IMUs’ small volume, easy-to-integrate characteristics.

A nine-axis IMU consists of a three-axis accelerometer, gyroscope and magnetometer, which measures linear accelerations, angular rates and local magnetic fields respectively. Using IMUs to estimate joint angles mainly follow a standard procedure. To be specific, firstly the raw IMU measurements are used to estimate the relative orientation of sensors with the reference of the Earth frame. The so-called “absolute orientations” of IMUs mounted on two adjacent limb segments are estimated following the methods proposed in inertial motion tracking-related literatures [19,20,21,22]. More precisely, the orientations of sensor-fixed coordinate frames with respect to a reference frame, i.e., the Earth coordinate frame, are estimated. Via the Earth frame, the relative orientation between two sensor-fixed frames can be estimated. Then, the sensor-to-segment alignment is performed by mapping the sensor-fixed coordinate frames with their respective segment-fixed coordinate frames. In this step, either calibration strategies including preset calibration postures [23,24,25] and calibration devices [26] or calibration-free methods [27] are employed to obtain the analytical axes like the biological joint rotational axis or the sensor-to-joint center axis and thus to build the alignment. Finally, a cascade multiplication of the rotation matrices estimated by the abovementioned two steps can be applied to get the relative orientation between the segment-fixed coordinate frames of two adjacent segments. Then we can estimate joint angles by decomposing the rotation matrix into rotations around each biological axis.

Among the steps of this standard procedure, an operation that aims to unify the estimated absolute orientations of different IMUs is reported with significant improvement of estimating accuracy [13,23,28,29,30], regardless of the degree of freedoms (DoFs) and methods. That is, the unifying operation compensates for the errors caused by the deviation of IMUs’ estimated reference frames. An ideal estimation of the absolute orientation can provide the relative orientation of an IMU’s sensor-fixed coordinate frame with respect to the Earth coordinate frame. However, due to the individual data characteristics of each IMU (e.g., local magnetic distortion, movement-caused corruption of acceleration and measurement noise), the estimated orientation of each sensor-fixed frame is actually depicted in different reference frames, rather than the Earth frame [31]. The unification-based operation, denoted by the reference frame unification, is to unify the different reference frames so that the sensor-fixed frames of the IMUs mounted on adjacent segments can be depicted in the same reference frame. Although there were studies of compensating for deviations of reference frames, to the best of our knowledge, the RFU problem was never systematically clarified and studied. Many issues, like the characteristics of the deviation, the suitable selection of different metrics that can be used to measure the deviations and the suitable RFU method that meets the characteristics of the deviation, still remain to be answered. Moreover, the performance of previously proposed methods is also limited by two aspects. Firstly, the metrics used to measure the deviation of IMUs’ reference frames are relatively too simple to provide an accurate and robust enough measurement. Pioneer studies like [23,28] utilized the consistency of the angular rates of one calibration motion to measure and compensate for the deviation. Following studies like [29,30] leveraged the joint axis of the hinge-joint constraint [27] to online detect the deviations and calculate the compensation. Some other studies [13] employed the consistency of the gravity acceleration. Secondly, the characteristics of the reference frames’ deviations should be systematically investigated so as to propose a suitable RFU method. The pioneer study [28] unified reference frames in a static manner. The following study [23] proposed a linear interpolated method that improve the RFU efficiency. Latter studies started to employ a point-wise compensation for RFU [13,29,30]. Although presenting increasing improvement, the compensation methods for RFU are proposed empirically without the instruction of reference frame deviation characteristics.

In this study, we systematically define the RFU problem and carefully distinguish it from the drift correction problem. Then, we analyze the deviations of reference frames caused by the different characteristics of IMUs, in the manner of excluding the confounding factors. Based on the analysis, we propose comprehensive metrics and further utilize it to form a novel RFU method that meets the characteristics of deviations. Both specially devised three-dimensional gimbal and human movement experiments are employed to separately estimate the performance. Performance comparison with previously used methods demonstrates the feasibility and improvement of our proposed method.

## 2. Related Work

Before presenting our method, it is important to firstly clarify some issues like what is the RFU problem, what is expected to know about the deviation of reference frames and how we get inspirations from literatures.

As stated above, reference frame unification is to let the sensor-fixed frames of IMUs depicted in the same reference frame, with the aim of decreasing the errors caused by deviations of reference frames and thus improving the accuracy of angle estimation. It should be noted that although most literatures confuse the “drift correction” [29,30,32,33] and the “reference frame unification”, RFU is significantly different from drift correction (DC). In our paper, we will systematically define the RFU problem, distinguish it with the DC problem and mathematically formulate them.

Previously used methods of calculating the rotational compensation of RFU are based on different hypotheses of reference frame deviations’ characteristics. Pioneer works like [23,28] assumed the time-varying characteristics of the deviations can be approximated statically or linearly. Following works utilized the consistency of the joint axes estimated by the hinge-joint constraint to calculate point-wise rotational compensation [29]. Although the performance is limited by the leveraged single metrics, the point-wise compensation theoretically provides a better approximation to the time-varying deviations. Other studies assumed the deviations can be statically compensated through fusing the corrections calculated by different metrics [31]. In our study, we experimentally study the time-varying characteristics of the deviations, and utilize the conclusions to propose a novel point-wise and time-varying compensation for RFU.

The metrics used to measure the deviations also relate to the calculation of the rotational compensation. Studies [23,28] calculated the rotational compensation based on the consistency of angular rates of IMUs mounted on thigh and shank during the hip abduction/adduction movement. The angular rate-based metric may be sensitive to the movement of other lower-limb joints. For example, if the knee rotated during the hip abduction/adduction movement, the consistency of the angular rates would be broken and would significantly degrade the RFU performance. Moreover, the implementation of calibration motions might impede the angle estimation technique’s broader usage. Ref. [29] proposed to measure the deviations by the joint axis of the hinge-joint constraint. Following the hinge-joint constraint, lower-limb joints were simplified as a 1-DoF joint, and the coordinates of the joint axis in sensor-fixed frames can be estimated. Through calculating the rotational deviation of the joint axis’ coordinates in different reference frames, the deviations of reference frames can be measured and then compensated. The joint axis-based metric might be limited by the hinge-joint constraint that simplifies the biological joints as 1-DoF joints [27]. Although the study [29] has proposed to screen the hinge-rotating case and interpolate the rotational compensation during non-hinge-rotating cases, as argued above, the linear interpolation still remains limitations. Fasel et al. [13] proposed to measure the deviations using the consistency of the gravity acceleration. That is, the accelerations measured by IMUs should process the same coordinates in the same reference frame. This metric, although promising, is limited by the corruption of movement-caused accelerations. In contrast to the single metric, Seel et al. [31] fused the rotational compensation calculated by the consistency of the local magnetic field and the gravity. This more comprehensive metric provided a better solution to decreasing the flaws of each single metrics, but was still impeded by the static fusion coefficient that needs manually tuning and the not-comprehensive-enough metrics. Inspired by the existing metrics and the lessons of their flaws, we propose a novel metric that comprehensively utilizes all the above-mentioned metrics, and thus contributes to a more accurate RFU.

## 3. Method and Materials

In this section, we firstly mathematically formulate the problem and secondly describe the experimental procedure and how the experiments are designed according to the paradigms of other works. Then, the experimental investigation is given in order to analyze the characteristics of reference frames’ deviations and to instruct the design of our RFU method. Finally, our RFU method is proposed by following the investigated characteristics and principles of reference frames’ deviations.

### 3.1. Problem Statement

**Figure 1:**
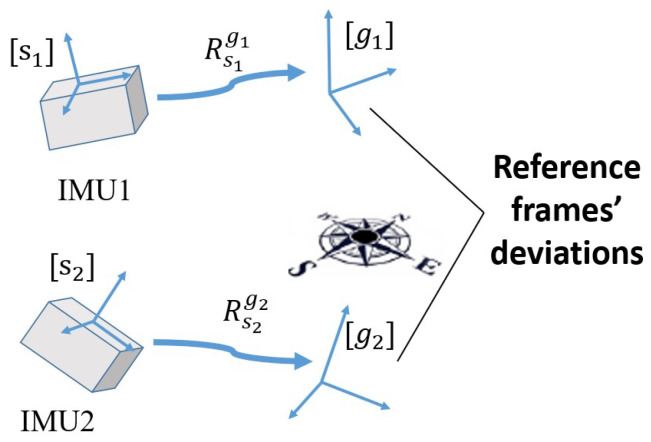
The schematic figure of reference frame unification, where $R_{s_{i}}^{g_{i}}$ denotes the rotaion matrix from the sensor-fixed frame $\left[s_{i}\right]$ to the reference frame $\left[g_{i}\right]$.

**Figure 2:**
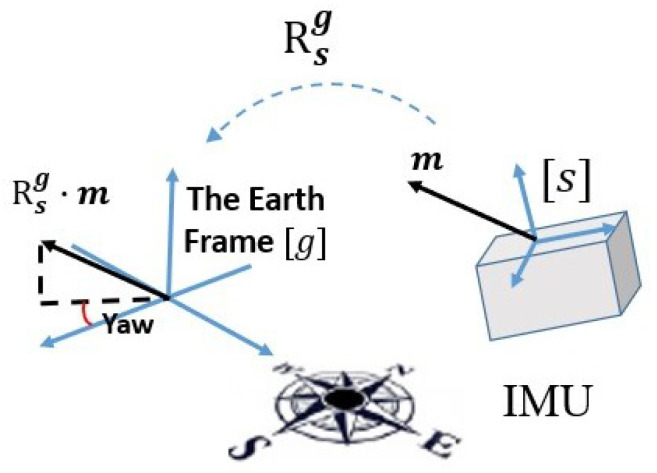
The schematic figure of drfit correction, where $R_{s}^{g}$ denotes the rotaion matrix from the sensor-fixed frame [s] to the Earth frame [g].

The RFU and DC problems can be mathematically formulated as follows. The measurements of the ith IMU can be described as ${\tilde{\omega }}_{i}=\omega_{i}+\delta_{\omega_{i}}$, ${\tilde{a}}_{i}=a_{i}^{\mathrm{move}}+g+\delta_{a_{i}}$, ${\tilde{m}}_{i}=m_{i}+\delta_{m_{i}}$, where g denotes gravity, δ denote noise and corruptions that differ across IMUs, ${\tilde{\omega }}_{i}$, ${\tilde{a}}_{i}$, ${\tilde{m}}_{i}$ denote measurements and $\omega_{i}$, $a_{i}$, $m_{i}$ denote real values of angular rate, movement-caused acceleration and local magnetic field. The absolute orientations estimated by the corrupted measurements are denoted by the rotation matrices $R_{s_{1}}^{g_{1}}$, $R_{s_{2}}^{g_{2}}$, which depict sensor-fixed coordinate frames’ orientations with respect to different reference frames. It should be noted that in the following, we treat the quaternion *q* and the rotation matrix *R* as equal and without stating the substitution between the two again. The RFU issue can be formulated as

(1) $$\begin{array}{c} q_{corr}=argmin\left(q_{s_{2}}^{g_{2}}⊗q_{corr}\left({\tilde{\omega }}_{i},{\tilde{a}}_{i},{\tilde{m}}_{i}\right)-q_{s_{1}}^{g_{1}}\right) \end{array}$$

where $q_{corr}$ denotes the rotational compensation of RFU. The DC issue can be formulated as:

(2) $$\begin{array}{c} q_{drift}=argmin\left(\left(q_{s_{i}}^{g_{i}}⊗q_{drift}\left({\tilde{\omega }}_{i},{\tilde{a}}_{i},{\tilde{m}}_{i}\right)\right)⊗r^{s_{i}}-r^{g_{i}}\right) \end{array}$$

where $q_{drift}$ denotes the rotational compensation of DC, $r^{s_{i}}$ and $r^{g_{i}}$ denote reference vectors in the sensor-fixed frame and the Earth frame, respectively. RFU can be significantly distinguished from DC from two aspects. On one hand, the result of RFU and DC refers to different coordinate frames. As shown in Figure 1 and Figure 2, RFU is to make the vectors in sensor-fixed frames depicted in the same reference frame, regardless of what the reference frame is. DC is to correct the drift of absolute orientation estimation mainly caused by magnetic distortion, expecting to make the reference frame the same as the Earth frame. On the other hand, RFU and DC differ by the calculation of the rotational compensation. Due to the magnetic measurements’ dominant influence of determining the azimuth angle of orientation, the operation of DC is to mainly calculate the rotational compensation of the horizontal plane. In contrast, RFU is just to rotate one reference frame to correspond to another one, without consideration of the rotational axis.

### 3.2. Experimental Procedure

**Figure 3:**
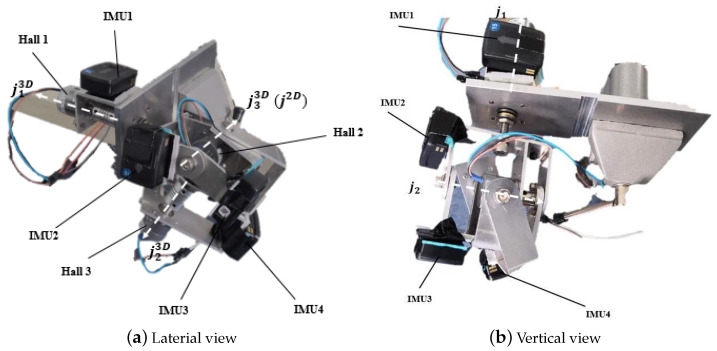
Laterial and vertical views of the 3-DoF gimbal.

In order to study the characteristics of different reference frames’ deviations, other factors during angle estimation, like sensor-to-body alignment, relative rotation between two segments, need to be decreased as much as possible. A gimbal with flat surfaces and known rotational axes was designed to mimic the biological lower-limb joints, mainly following the paradigm of the design in [23]. As shown in Figure 3, the Hall sensors attached to each axis by couplings were used to measure angles of each rotational axis, the signals of which were sampled at 500 Hz. Four IMUs (74Hz, Delsys Trigno, IM type & Avanti type) were attached to each segment of the gimbal. Only IMU2 and IMU3, which were attached to the segments beside a 1-DoF joint (the joint axis $j_{2}$), were used in this study. **Z** axis of IMU2 and **X** axis of IMU3 were placed in the direction of the main axis $j_{2}$. The null position of the axis $j_{2}$ is to let the **X**-**Z** plane of IMU2 and **Y**-**Z** plane of IMU3 stay in the same plane, which is ensured by the devised mechanical structure of the gimbal. Due to the flat mounting surface, IMUs can be mounted with known orientation relative to segment-fixed frames by the means of manually aligning the sides of IMUs and gimbal following the manners of [23,34]. In this way, errors of the sensor-to-segment alignment can be maximally excluded from the calculation. The influence of relative rotation between two segments, i.e., the mounting surfaces besides the joint axis $j_{2}$, will be excluded by calculation stated in latter sections.

In order to evaluate the superiority of our proposed RFU method, level walking experiments on human subjects were performed. Ten healthy subjects without previous neuropathological history (7 males and 3 females, age range: 20–30 years, height range: 155–184 cm, weight range: 50–90 kg) were recruited and asked to perform five trials of 3-min level walking. Rest periods were allowed between trials to avoid fatigue. The experiment protocol was approved by the local ethical committee and all participants had been informed of the content and their right to withdraw from the study at any time, without giving an explanation.

**Figure 4:**
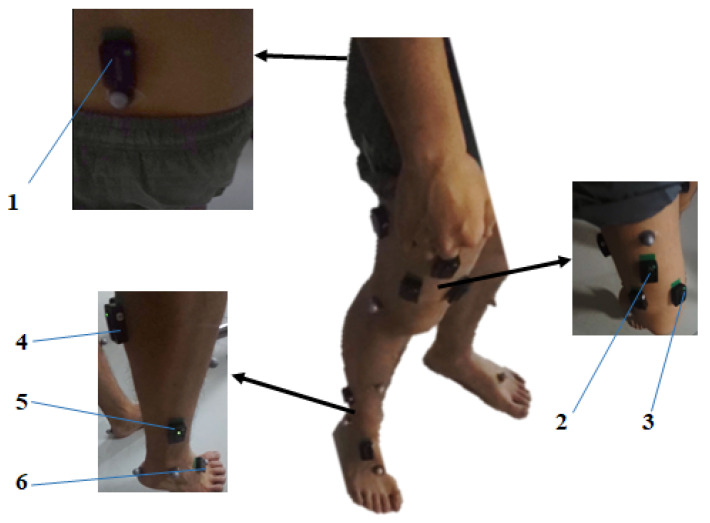
The sensor attachment on subjects. The numbers 1–6 denote the IMUs placed on the lower back, the thigh, the shank and the foot, repspectively.

Before and after the five trials, calibration postures proposed in [25] were asked to be performed by each subject in order to calculate the 3-dimensional joint angles of each joint. Besides, the hip abduction/adduction movement, following the paradigm of [23,28], was also performed in order to calculate the static and linearly interpolated RFU based on the angular-rate metrics. As shown in Figure 4, multiple IMUs were attached to the trunk, right thigh, right shank and right foot. The redundant IMUs were attached to study the influence of IMUs’ placement, which will feature in our future study. In this work, we randomly select one IMU per segment without loss of generality. Given that we employ magnetometer measurements in IMUs’ orientation estimating, ferromagnetic metal plates were placed in the proximity of IMUs in order to induce local magnetic distortion, which followed the paradigm of [30]. Sixteen retro-reflective markers were attached to subjects’ pelvis and lower limbs following the principles of [14,15], whose 3-D locations were recorded (100 Hz) using an 8-camera video system (Vicon, Oxford, UK). The joint angles were calculated by the pose estimation and inverse kinematics model embedded in the software Visual 3D. The signals from nine-axis IMUs and the video system were synchronized by the trigger and time stamps.

### 3.3. Analysis on the Characteristics of Different Reference Frames’ Deviations

#### 3.3.1. Data Preprocessing

**Figure 5:**
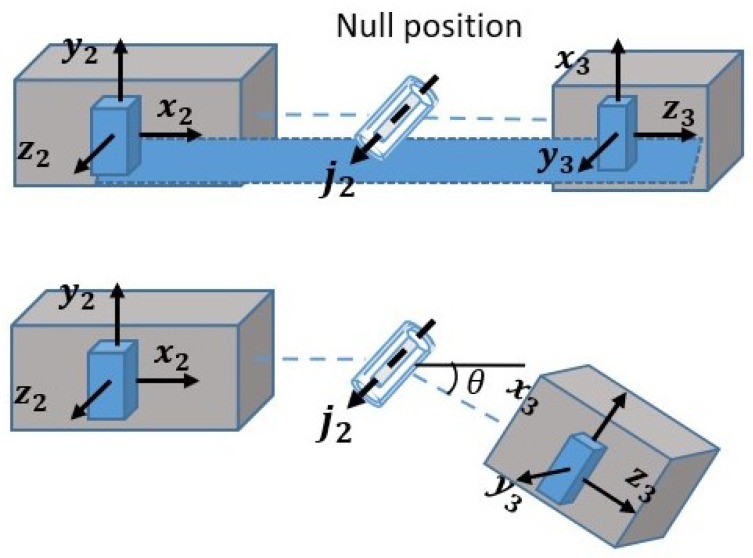
The schematic diagram of the rotational relationship around the axis $j_{2}$ of the gimbal.

In order to evaluate the deviations’ characteristics of different IMUs, primary factors like different angular rates and IMUs’ individual characteristics should be included, while confounding factors like sensor-to-body alignment, rotation-caused IMUs’ orientation difference should be excluded. To be specific, confounding factors can be excluded by the careful alignment of IMUs and the gimbal’s mounting surface and some calculations. As shown in Figure 5, on the null position, the **Z** axis of the sensor-fixed frame of IMU2, the **Y** axis of the sensor-fixed frame of IMU3 and the axis $j_{2}$ are in the same direction, and the **X**-**Z** plane of IMU2 corresponds to the **Y**-**Z** plane of IMU3. The determination of the sensor-fixed frames of IMU2 and IMU3 is adopted from the user guide of Delsys. Under this circumstance, the rotation matrix between the sensor-fixed frames of IMU2 and IMU3 can be given as

(3) $$T_{IMU2}^{IMU3}=\left[\begin{array}{ccc} 0 & 1 & 0\\ 0 & 0 & 1\\ 1 & 0 & 0 \end{array}\right]$$

where $T_{IMU2}^{IMU3}$ denotes the rotation that transform the orientation of the sensor-fixed frame of IMU2 to that of IMU3. If the axis $j_{2}$ is manually rotated away from the null position, as shown in Figure 5, the consequent rotation can be depicted by the matrix

(4) $$R=\left[\begin{array}{ccc} \cos\theta & 0 & -\sin\theta \\ 0 & 1 & 0\\ \sin\theta & 0 & \cos\theta \end{array}\right]$$

where *R* denotes the rotation matrix, θ denotes the rotational angle around the axis $j_{2}$, measured by the Hall sensor. In so doing, the orientation of the sensor-fixed frame of IMU2 can be transformed into that of IMU3 by the cascade multiplication of $T_{IMU2}^{IMU3}$ and *R*. Thus, confounding factors are excluded. As for the primary factors, the manually activated rotation around $j_{2}$ contributes to different angular rates of the two IMUs thus affects the absolute orientation estimating of both IMUs, while employing two different IMUs also contributes to the different IMUs’ individual characteristics.

We next implement the absolute orientation estimating methods adopted from [22] in order to estimate the absolute orientations of IMU2 and IMU3. The estimates are denoted by rotation matrices, i.e., $R_{s_{2}}^{g_{2}}$ for the absolute orientations of IMU2 and $R_{s_{3}}^{g_{3}}$ for absolute orientations of IMU3. The estimation accuracy denoted by the root mean square error (RMSE) during dynamic movement was reported to be 1.63 deg in [22]. Following the calculations of excluding the confounding factors, the transformed orientation of IMU2 is calculated as follow.

(5) $$\begin{array}{c} {\tilde{R}}_{s_{2}}^{g_{2}}=R\cdot T_{IMU2}^{IMU3}\cdot R_{s_{2}}^{g_{2}} \end{array}$$

#### 3.3.2. Analyzing Procedure

An analysis is performed on the orientation deviations of the two IMUs. Firstly, XYZ Euler angle decomposition is performed to transform the estimated rotation matrices, ${\tilde{R}}_{s_{2}}^{g_{2}}$ and $R_{s_{3}}^{g_{2}}$, into Euler angles, which are denoted by $\alpha_{i}$, $\beta_{i}$, $\gamma_{i}$ for **X**, **Y**, **Z** axes, *i* = 2, 3 for IMU2 and IMU3 respectively. Secondly, differences of each Euler angles, denoted by Δα, Δβ, Δγ, are calculated for further analysis. Thirdly, overall deviations and time-evolving characteristics are depicted and analyzed by RMSE and statistical analysis. Particularly, the differences obtained in each trial are divided into three subsets, each lasting 40 s. Then the RMSEs of each subset are averaged over all the five trials. One-way ANOVA is employed to assess the results.

#### 3.3.3. Analyzing Results

In the following, we report our primary findings of the deviations and our analyzing results. Then, we discuss the findings related to the deviations, expecting to indicate the design of our method.

**Figure 6:**
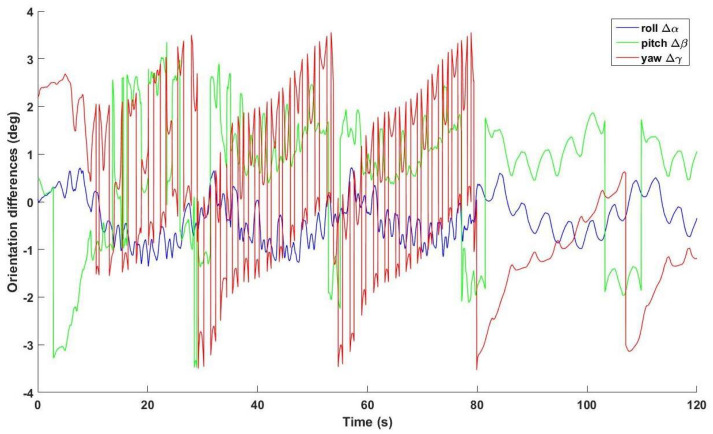
Representative plots of deviations depicted by Euler angle differences.

**Figure 7:**
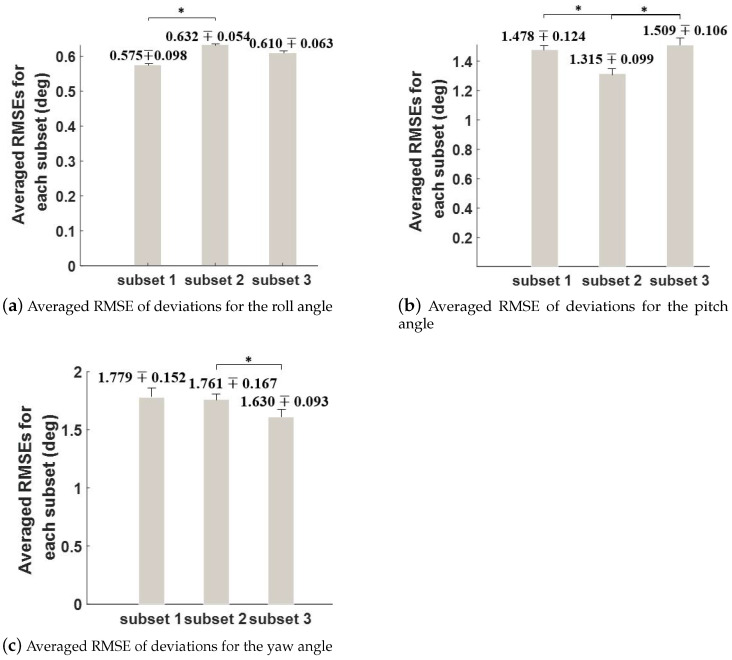
Analysis on RMSEs of Euler angle differences. * denotes the statistical difference.

As shown in Figure 6, the differences of the estimated absolute orientations of IMU2 and IMU3 are depicted by the differences of XYZ-decomposed Euler angles. The ranges of Δα, Δβ, Δγ are 2.071 deg, 6.836 deg and 7.082 deg, respectively. The relative RMSEs are presented in Figure 7. As shown in Figure 7a, the RMSEs for the roll angle of the three subsets averaged over all the trials are presented. There is a significant difference between the RMSEs of the first two subsets. As shown in Figure 7b, the RMSEs for the pitch angle of the three subsets averaged over all the trials are presented. There is a significant difference between the RMSEs of the first two subsets and between those of the last two subsets. As shown in Figure 7c, the RMSEs for the yaw angle of the three subsets averaged over all the trials are presented. There is a significant difference between the last two subsets in term of RMSE.

#### 3.3.4. Findings

Our main findings basically support our statement in the related work session. That is, (1) the deviations non-linearly evolve over time; (2) the deviations between reference frames are significantly different from the drifts caused by magnetic distortion; (3) the deviations significantly differ from IMUs’ estimation drift, especially from heading drift.

Firstly, the significant differences existed among RMSEs of each subset indicate the time-varying characteristics of the deviations. Thus, the static method proposed in [28] cannot provide an acceptable compensation for the deviations, i.e., an accurate unification of reference frames, which meet the conclusion of [23].

Secondly, the time-dependent varying of deviations is non-linear. As shown in curves of Figure 6, the deviations do not vary in a linear or pseudo-linear way. Rather, a combined partially piecewise linear and non-linear manner is presented. This phenomenon suggests that the deviations can be compensated in a piecewise linear way.

Thirdly, heading drift is obviously not the only origin of the deviations. As stated in the related work, most of the related literatures confuse the “drift correction” with the “reference frame unification”. That is, the deviations of reference frames are attributed to the heading drift caused by the local magnetic distortion. As shown in Figure 7a,b, the deviations on the pitch and roll angle present a significant amount compared with that of the deviations on the yaw angle. Other than the heading drift that primarily affects the accuracy of yaw, the deviations of the two reference frames, even when confounding factors are excluded, still include estimates’ differences on pitch and roll resulted from other factors, such as the movement-caused corruptions of accelerations and measurement noise. Moreover, the RMSEs and range of deviations reported in our analyzing results are relatively larger than those reported in previous IMU orientation estimating studies [21,22]. This also gives side proof of the distinction of “estimation drift” and “reference frame deviation”. The larger results of our study result from the combined usage of estimation drift and individual characteristics of IMUs’ measurements.

In summary, the findings indicate that the RFU method should not only compensate for the estimates’ heading drift but also take other factors into consideration. Moreover, pointwise RFU should be performed to meet the non-linearly time-varying characteristics of the deviations, if possible, with multiple metrics to give a comprehensive measurement.

### 3.4. Reference Frame Unification with Comprehensive Metrics

**Figure 8:**
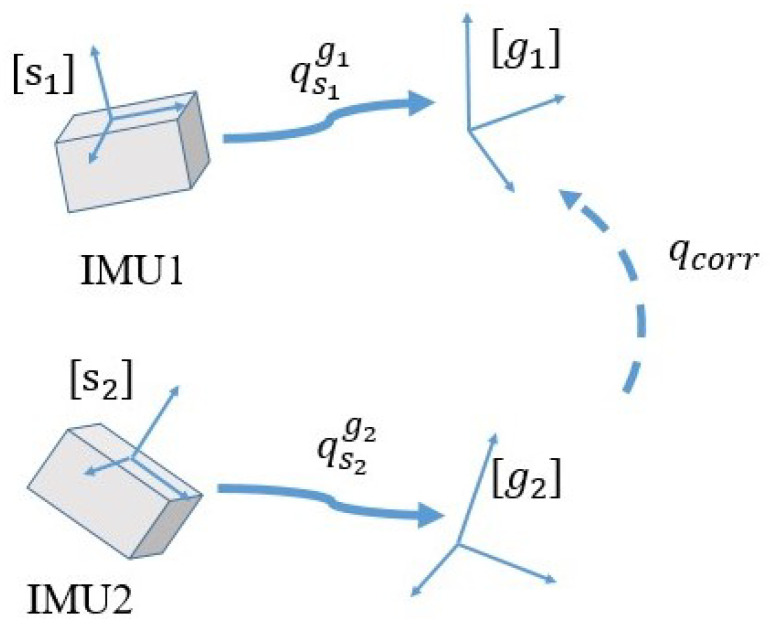
Schematic figure of correction quaternion for the reference frames’ deviations, where $\left[g_{1}\right]$, $\left[g_{2}\right]$ denote reference frames estimated by IMU1’s and IMU2’s measurements.

As stated in the problem statement and Figure 8, the RFU issue is to find and calculate a correction quaternion $q_{corr}$ that rotates $\left[g_{2}\right]$ to $\left[g_{1}\right]$, thus both absolute orientations are depicted in the same reference frame. Indicated by the findings and literatures, in this section, we propose a novel RFU method that can provide a pointwise compensation with comprehensive metrics. Our method is distinguished from other RFU methods by (1) comprehensively using magnetic fields, accelerations and the coordinates of the main axis as metrics and (2) point-wise compensation of the non-linearly time-varying reference frames’ deviations.

In order to calculate $q_{corr}$, some measurements that are common among reference frames and sensor-fixed coordinate frames should be employed as an intermediate reference to solve the RFU issue. Inspired by literatures, the gravity acceleration, the local magnetic field and the joint axis under the hinge-joint constraint can be leveraged as the intermediate reference. Without considering the movement-caused accelerations, the acceleration vectors, $a_{1}$ and $a_{2}$, should be identical in the common reference frame, which contributes to the construction of the correction quaternion, given by

(6) $$\begin{array}{cc} q_{acc} & =\cos\left(\theta_{acc}/2\right)\cdot \left[\begin{array}{c} 1\\ 0\\ 0\\ 0 \end{array}\right]+\sin\left(\theta_{acc}/2\right)\cdot \left[\begin{array}{c} 0\\ W_{\mathrm{acc}} \end{array}\right]\\ W_{\mathrm{acc}} & =\left(q_{s_{1}}^{g_{1}}⊗a_{1}\right)\times \left(q_{s_{2}}^{g_{2}}⊗a_{2}\right)\\ \theta_{acc} & =\frac{q_{s_{1}}^{g_{1}}⊗a_{1}\times q_{s_{2}}^{g_{2}}⊗a_{2}}{∥\left(q_{s_{1}}^{g_{1}}⊗a_{1}\right)\times \left(q_{s_{2}}^{g_{2}}⊗a_{2}\right)∥} \end{array}$$

where $W_{\mathrm{acc}}$ is the rotation axis, $\theta_{acc}$ is the rotation angle and $q_{acc}$ is the correction quaternion calculated from gravity. Similarly, the identical magnetic field vectors $m_{1}$ and $m_{2}$ without magnetic distortion could contribute to another correction quaternion, given by

(7) $$\begin{array}{cc} q_{mag} & =\cos\left(\theta_{mag}/2\right)\cdot \left[\begin{array}{c} 1\\ 0\\ 0\\ 0 \end{array}\right]+\sin\left(\theta_{mag}/2\right)\cdot \left[\begin{array}{c} 0\\ W_{\mathrm{mag}} \end{array}\right]\\ W_{\mathrm{mag}} & =\left(q_{s_{1}}^{g_{1}}⊗m_{1}\right)\times \left(q_{s_{2}}^{g_{2}}⊗m_{2}\right)\\ \theta_{mag} & =\frac{q_{s_{1}}^{g_{1}}⊗m_{1}\times q_{s_{2}}^{g_{2}}⊗m_{2}}{∥\left(q_{s_{1}}^{g_{1}}⊗m_{1}\right)\times \left(q_{s_{2}}^{g_{2}}⊗m_{2}\right)∥} \end{array}$$

However, due to the corruption of acceleration and magnetometer readings, neither of the correction quaternions calculated above is accurate enough to represent the rotational relationship between $\left[g_{1}\right]$ and $\left[g_{2}\right]$. Herein, a weighted sum of these two correction quaternions is used to make a fusion.

(8) $$\begin{array}{c} q_{corr}=k_{mag}\cdot q_{mag}+k_{acc}\cdot q_{acc} \end{array}$$

In [31], Seel et al. proposed to fuse the two correction quaternions by manually tuning the coefficients. The manually tuned fusion coefficients, as argued in our previous work [22], cannot well fit the time-varying trends of the corruptions of movement-caused accelerations and magnetic distortion. Thus, dynamic tuning should be included. Inspired by the works [29,30] that solely used the joint axes estimated by the hinge-joint constraint, we employ the same paradigm here to form a more comprehensive metric. To be specific, $j_{1}$ and $j_{2}$, denoted as the coordinates of the joint axis of a hinge joint depicted in the two sensor-fixed coordinate frames, are estimated by the method proposed in [27]. After estimating the absolute orientations, the deviation of ${\left[j_{1}\right]}_{g_{1}}$ and ${\left[j_{2}\right]}_{g_{1}}$ is introduced here to evaluate to what extent coordinates in $\left[g_{2}\right]$ is rotated by $q_{corr}$ into those in $\left[g_{1}\right]$, where ${\left[j_{1}\right]}_{g_{1}}$ and ${\left[j_{2}\right]}_{g_{1}}$ are $j_{1}^{2D}$ and $j_{2}^{2D}$ described in the reference frame $\left[g_{1}\right]$,given by

(9) $$\begin{array}{cc} {\left[j_{1}\right]}_{g_{1}} & =q_{s_{1}}^{g_{1}}⊗j_{1}\\ {\left[j_{2}\right]}_{g_{1}} & =q_{s_{2}}^{g_{2}}⊗q_{corr}⊗j_{2} \end{array}$$

where ⊗ denotes the multiplication of quaternions. Regarding $f_{i}\left(k_{mag},k_{acc}\right)=∥{\left[j_{1}\right]}_{g_{1}}-{\left[j_{2}\right]}_{g_{1}}∥$ as the cost function, fusion coeficients, $k_{mag}$ and $k_{acc}$, can be estimated through the secant version of L-M method. Hence, the calibration of reference frames synthesizes the information from local magnetic field, gravity and the main axis of the 3-DOF joint, which results in a comprehensive metric.

**Figure 9:**
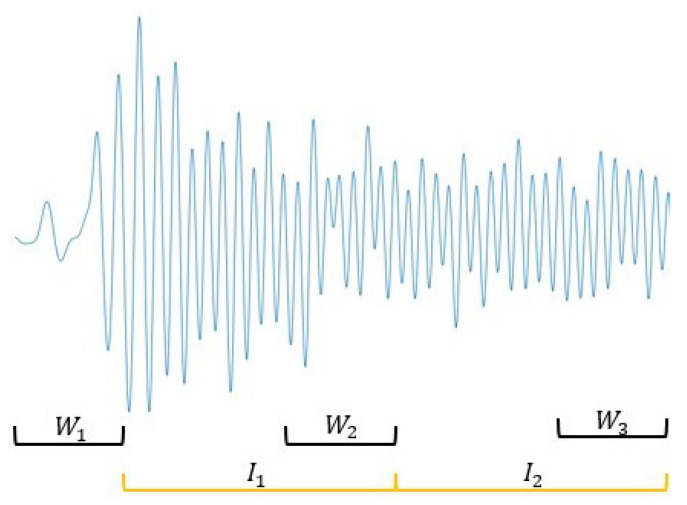
Data windowing scheme.

For the sake of real-time calculation, as shown in Figure 9, two sliding windows are separated by an interval ($I_{n}$) within which the fusion coefficients estimated in the last sliding window are used to unify reference frames in the interval. At the end of an interval, the last N measurements could be used to compute the cost function and execute iterations to update fusion coefficients.

### 3.5. Data Analysis

Data recorded in the experiment on human subjects are analyzed as follow. Firstly, accuracy is depicted by RMSE between IMU-calculated joint angles and the angles measured by the optical motion capture system, for 3-dimensional angles of hip, knee and ankle. RMSEs of the five trials are averaged, from which the means and standard deviations (SD) are derived in order to evaluate the accuracy and precision. In addition, one-way ANOVA is applied to the performance of different RFU methods. Secondly, in order to evaluate the influence of movements on the correction quaternion, the repeatability test is performed by the dispersion of the five correction quaternions, following the paradigm of [13]. Particularly, the dispersion χ of the five correction quaternions $q_{S,T}$ is calculated around their mean $q_{S}$ for all subjects.

(10) $$\begin{array}{c} \chi =\sqrt{\frac{1}{S*T-1}\cdot \sum_{S,T}\Delta_{S,T}^{2}} \end{array}$$

(11) $$\begin{array}{c} \Delta_{S,T}=2\cos(∥q_{S}⊗q_{S,T}{∥}_{real}) \end{array}$$

where $\Delta_{S,T}$ corresponds to the orientation angle difference between $q_{S}$ and $q_{S,T}$. *S* denotes the athletes 1, …, 10, *T* the trials 1, …, 5, and the quaternion multiplication.

## 4. Results

Performance is analyzed following the paradigm in the data analysis section. For the comparison purpose, we implement four previously used RFU methods, which are the static [28] and linearly interpolated [23] methods supported by the hip abduction/adduction before and after measurement, the joint axis-based pointwise RFU method [29] and gravity acceleration-based method [13]. All the data are processed by MATLAB 2015 B.

**Figure 10:**
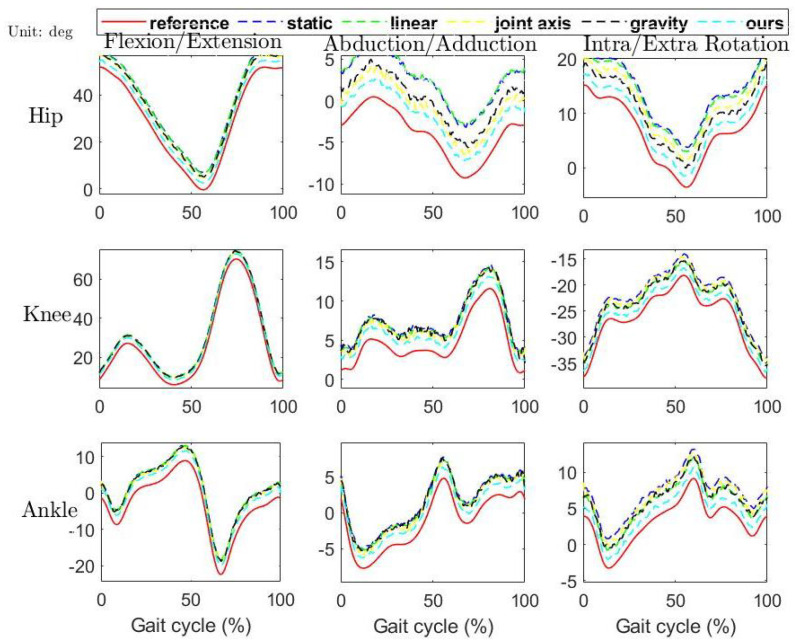
Sample plots of the 3-dimensional joint angle curves estimated by employing all the five RFU methods, where “static” denotes the static RFU method [28], “linear” denotes the linearly interpolted RFU method [23], “joint axis” denotes the joint axis-based RFU method [29], “gravity” denotes the gravity acceleration based method [13] and “ours” denotes the RFU method proposed in this paper.

**Figure 11:**
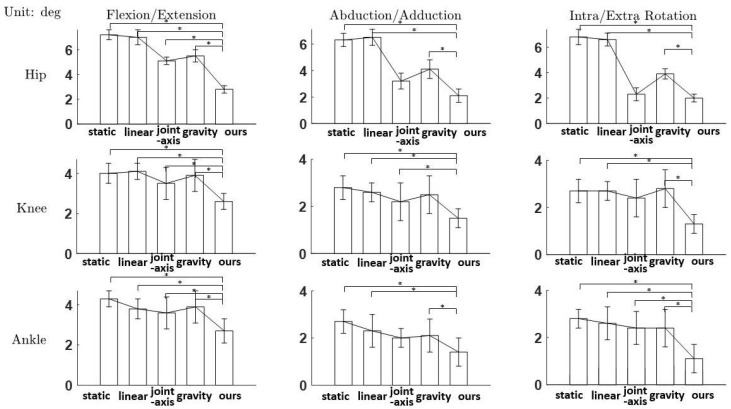
RMSEs of each joint and each dimension averaged over trials and subjects, where “static” denotes the static RFU method [28], “linear” denotes the linearly interpolted RFU method [23], “joint axis” denotes the joint axis-based RFU method [29], “gravity” denotes the gravity acceleration based method [13] and “ours” denotes the RFU method proposed in this paper, * denotes the significant difference analyzed by the one-way ANOVA (α < 0.05).

As shown in Figure 10, all the estimates track the “standard” curves well with similar trends. As shown in Figure 11, our proposed RFU method significantly outperforms other RFU methods (α<0.05), which are depicted by the means and standard deviations of RMSEs. The RMSEs of each RFU methods averaged over trials and subjects are shown in Table 1.

As shown in Table 2, the dispersion of correction quaternions of each RFU method range from 2.37 deg to 4.37 deg. The correction quaternion of linear interpolated RFU method presents the smallest dispersion, while the correction quaternion of joint axis-based RFU method presents the largest.

### Discussion

This paper sought to firstly formulate the issue of reference frame unification and distinguish it with drift correction, and secondly to design, implement and evaluate a novel RFU method with comprehensive metrics based on the experimental analysis of the reference frames’ deviations. We evaluate our method by estimating human lower-limb joints from IMUs’ measurements in the specially designed heterogeneous magnetic field and compare the performance with conventional RFU methods. The RMSEs of 3-dimensional angle estimation that uses our RFU method are all under 3 deg, which is significantly better than the accuracy of using other methods. The standard deviations and dispersions of our method present similar repeatability with other methods, which as discussed in [23] suggests its promising application on clinical usage. These findings indicate that our proposed RFU method fits better to the time-varying characteristics of reference frames’ deviations, thus measures and compensates for the deviations with state-of-the-art accuracy.

The accuracy of other RFU methods reported in our work is generally larger than that reported in literatures. The RMSEs on knee angles of the static, linearly interpolated and joint axis-based methods are all relatively higher than those reported in their original papers. This might result from two aspects. On one hand, as stated above, our experiments are conducted in the heterogeneous magnetic field, which as indicated in the study [30] significantly increase the estimation errors. On the other hand, studies of some literatures are performed using mechanical gimbals, as discussed in [23,34], the performance on the gimbal is different from that on human subjects, especially considering the fact that biological joints are neither ideal hinge nor screw joint. The RMSEs are also larger than those reported in [25] from which we employed the functional calibration postures. Although it is reported that the sensor-to-body alignment is magnetic distortion-free, the sensor-to-reference frame orientation is sensitive to the magnetic distortion and other factors like movement-caused accelerations’ corruption. In addition, the accuracy of the gravity acceleration-based method reported in our work is similar to that reported in its original work [13]. This is because our experiment is conducted for level walking, while the study [13] is conducted for skiing, which presents a larger range of motion and movement-caused accelerations.

The accuracy of our proposed RFU method is significantly better than that of others. The reason could be twofold. Firstly, as indicated by our analysis of the reference frames’ deviations, the RFU method is supposed to be able to compensate for the non-linearly time-varying deviations. That is, the RFU method would better be pointwise. Compared with the static and linearly interpolated methods, our proposed method provides a pointwise compensation and the fusion coefficient is time-varied in each interval. Secondly, the metrics used to measure the deviations are more comprehensive than the metrics used in other methods. Compared with the joint axis-based and gravity acceleration-based methods that are also pointwise, our method’s better performance demonstrates the efficiency of utilizing the more comprehensive metrics so that effects of the corruptions of each single metric can be decreased.

The repeatability and precision depicted by the dispersions and standard deviations present similarity with the data reported in literatures. The dispersions of the gravity acceleration-based method are slightly lower than that of its original study. It is because, as stated in [13], the original study performed on skiing results in a greater dispersion than normal lower-limb motions, like level walking. As for the dispersion of the joint axis-based RFU method, it is surprising that its dispersion is larger than those of static and linear methods, although it meets the larger precision reported in [29]. As discussed in [29], the hinge-joint constraint cannot always be satisfied, especially for the hip that is reported with greater similarity to the screw joint. The relatively larger dispersion and precision of our proposed method might result from the involvement of the gravity acceleration and joint axis.

The current study has several limitations. Firstly, performance is just evaluated on functional calibration-based sensor-to-body alignment and the accuracy is thus limited by the employed aligning method. There is an alternative way of aligning sensors to body segments without employing calibrations [27]. Further tests will be performed to evaluate the performance on such aligning methods. Secondly, the performance is also limited by the absolute orientation estimating performances of the Algorithm 1 we employed [22]. The reported performance only reflects the RFU method-caused accuracy improvement on this algorithm. Thirdly, performance is just evaluated on level walking. More motion patterns should be included for future work.
**Algorithm 1** RFU method with comprehensive metrics.**Require:**$\omega_{1},\omega_{2},a_{1},a_{2},m_{1},m_{2},j_{1},j_{2},q_{s_{1}}^{g_{1}},q_{s_{2}}^{g_{2}}$**Ensure:**$k_{mag},k_{acc},q_{corr}$ 1: initialize $k_{mag},k_{acc}$ 2: **while**
sliding
windows
**do** 3:  calculating the magnetometer measurement-based correction quaternion $q_{mag}$ using Equation (7) 4:  calculating the accelerometer measurement-based correction quaternion $q_{acc}$ using Equation (6) 5:  fusing $q_{mag}$ and $q_{acc}$ using Equation (8) 6:  calculating the main axis’ projections in the reference frame $\left[g_{1}\right]$ using Equation (9) 7:  forming the cost function as $f_{i}\left(k_{mag},k_{acc}\right)=∥{\left[j_{1}\right]}_{g_{1}}-{\left[j_{2}\right]}_{g_{1}}∥$, and solving it using L-M method. 8: **end while**

## 5. Conclusions

In this study, we clarify and formulate the issue of reference frame unification and propose a novel RFU method with a more comprehensive metric based on the analysis of the time-varying characteristics of reference frames’ deviations. The analysis indicates the non-linear time-varying characteristics of deviations and the RFU’s difference with drift correction, which further instructes the design of our method. The proposed method, which unifies the reference frames with a more comprehensive metric in a pointwise manner, is validated by the results. Specifically, the RMSEs of our method (2.7 deg, 1.4 deg, 1.1 deg for ankle, 2.6 deg, 1.5 deg, 1.3 deg for knee and 2.8 deg, 2.1 deg, 2.0 deg for hip) are significantly smaller than those of previously used RFU methods. These performances indicate that our method, even in the heterogeneous magnetic field, can achieve acceptable accuracy. The repeatability depicted by dispersion also demonstrates our method can be used across multiple usages with robust performances. This work would assist in further improving the accuracy of IMU-based joint angle estimation.

## Figures and Tables

**Table 1:** RMSEs of each RFU methods.

		Static	Linear	Joint Axis	Gravity	Ours
Ankle	flexion/extension	4.3 ± 0.4	3.8 ± 0.5	3.6 ± 0.8	3.9 ± 0.8	2.7 ± 0.6
	abduction/adduction	2.7 ± 0.5	2.3 ± 0.7	2.0 ± 0.4	2.1 ± 0.7	1.4 ± 0.6
	intra/extra rotation	2.8 ± 0.4	2.6 ± 0.7	2.4 ± 0.7	2.4 ± 0.8	1.1 ± 0.6
Knee	flexion/extension	4.0 ± 0.5	4.1 ± 0.4	3.5 ± 0.8	3.9 ± 0.8	2.6 ± 0.4
	abduction/adduction	2.8 ± 0.5	2.6 ± 0.4	2.2 ± 0.8	2.5 ± 0.8	1.5 ± 0.4
	intra/extra rotation	2.7 ± 0.5	2.7 ± 0.4	2.4 ± 0.8	2.8 ± 0.8	1.3 ± 0.4
Hip	flexion/extension	7.2 ± 0.4	7.0 ± 0.6	5.1 ± 0.3	5.5 ± 0.5	2.8 ± 0.3
	abduction/adduction	6.3 ± 0.5	6.5 ± 0.6	3.2 ± 0.6	4.1 ± 0.7	2.1 ± 0.5
	intra/extra rotation	6.8 ± 0.6	6.6 ± 0.5	2.3 ± 0.5	3.9 ± 0.4	2.0 ± 0.3

**Table 2:** Dispersion of the calibration quaternions.

Static	Linear	Joint Axis	Gravity	Ours
2.56	2.37	3.74	4.37	2.89

## Abbreviations

The following abbreviations are used in this manuscript:

IMU	Inertial measurement unit
RFU	Reference frame unification
DC	Drift correction
